# Motor skill learning induces brain network plasticity: A diffusion-tensor imaging study

**DOI:** 10.1371/journal.pone.0210015

**Published:** 2019-02-06

**Authors:** Yan-Ling Pi, Xu-Heng Wu, Feng-Juan Wang, Ke Liu, Yin Wu, Hua Zhu, Jian Zhang

**Affiliations:** 1 Shanghai Punan Hospital of Pudong New District, Shanghai, China; 2 Key Laboratory of Exercise and Health Sciences of Ministry of Education, Shanghai University of Sport, Shanghai, China; 3 Physical Education and Educational Science Department, Tianjin University of Sport, Tianjin, China; University of Texas at Austin, UNITED STATES

## Abstract

Motor skills and the acquisition of brain plasticity are important topics in current research. The development of non-invasive white matter imaging technology, such as diffusion-tensor imaging and the introduction of graph theory make it possible to study the effects of learning skills on the connection patterns of brain networks. However, few studies have characterized the brain network topological features of motor skill learning, especially open skill. Given the need to interact with environmental changes in real time, we hypothesized that the brain network of high-level open-skilled athletes had higher transmission efficiency and stronger interaction in attention, visual and sensorimotor networks. We selected 21 high-level basketball players and 25 ordinary individuals as control subjects, collected their DTI data, built a network of brain structures, and used graph theory to analyze and compare the network properties of the two groups at global and regional levels. In addition, we conducted a correlation analysis on the training years of high-level athletes and brain network nodal parameters on the regional level to assess the relationship between brain network topological characteristics and skills learning. We found that on the global-level, the brain network of high-level basketball players had a shorter path length, small-worldness, and higher global efficiency. On the regional level, the brain nodes of the high-level athletes had nodal parameters that were significantly higher than those of control groups, and were mainly distributed in the visual network, the default mode network, and the attention network. The changes in brain node parameters were significantly related to the number of training years.

## Introduction

A large number of studies have found that motor skill training and acquisition can cause brain plasticity [1–4]. These changes involve the optimization of the working pattern of local brain regions as well as global brain network connectivity [5]. Using noninvasive neuroimaging techniques such as functional magnetic resonance imaging (fMRI) [6] and diffusion-tensor imaging (DTI)[7], it is possible to detect structural and functional brain plasticity after long-term motor skill training and acquisition. Understanding the neural mechanisms underpinning this plasticity may provide a basis for determining the types of practice or training that are most beneficial for enhancing performance [8]. Thus, the study of plastic changes associated with skill learning and expertise in the human brain is one of the most pertinent areas of current neuroscience research.

Previous voxel-based studies have reported that motor skill training and acquisition can induce changes in the structural and functional properties of specific brain areas that are involved in a practiced task [3, 4, 9–11]. In recent years, the introduction of DTI and graph theory has led to the conceptualization of the gross organization of the human brain as a structural network of connections comprising the “connectome” [12, 13]. The “connectome” models different brain areas by constructing networks with mathematical structures (such as nodes and edges] and reveals the architectural properties of the nervous system. In contrast to voxel-based approaches that focus on the local changes in various brain areas, graph theory tests the organizational structure and interactions among these brain areas at the system level, thus emphasizing the topological properties of the brain network as a whole [14, 15]. A pioneering study using graph theory to examine the brain networks of elite athletes was conducted by Wang et al. (2013) [16]. Wang and colleagues reported that the density of connections among the sensorimotor, attentional, and default-mode systems in the brain networks of elite word class gymnasts were increased. However, different types of motor skill learning lead to different forms of brain plasticity. Based on the extent to which the environment is stable and predictable during performance, motor skills can be classified into closed or open skills [17]. The study by Wang et al. revealed the effects of closed skill learning on brain networks, since a gymnastic routine is a typical closed skill. To date, the effect of open skill learning on brain network connectivity remains unclear.

In contrast to closed skills, an open skill is one that is performed in an environment that is variable and unpredictable. In these situations, the performer must use the processes of perception and decision-making to adjust his/her movements in response to changing environmental conditions, often in a short amount of time [17]. Excellent visual perception is the basis of effective prediction of the future moves of others (and hence, the future responses to them) [18]. Behavioral research on visual tasks for open skilled experts (such as in action games, basketball, volleyball, and water polo) have suggested that the experts outperform their peers on multiple-object tracking, visual search, and perceptual prediction [19]. Neuroimaging researches on similar subjects have also found greater activity in visual-related regions in the brain such as the intraparietal sulcus [20], indicating the different modes of brain activity. Considering the network-based processes in the brain, it is reasonable to assume that at the network level, the visual-related brain regions play an important role in transmission of information.

Consequently, we selected experts of a typical open skill (basketball) as subjects and reconstructed the structural networks of brain white matter. We then adopted graph theory to analyze brain network changes. We compared the global and regional measurements in athletes and controls, and examined the correlation between the changes in brain networks and the degree of motor skills. Subsequently, we determined the regions related to the experience of open skill learning. We hypothesized that compared to controls, the network importance of areas in the sensorimotor, attentional, and fault-mode network in open skilled experts would have been improved.

## Materials & methods

### Subjects

We studied 22 basketball players (mean age 20.65 ± 1.4 years) and 21 controls (mean age 22.59 ± 1.7 years). The age analysis was performed using a two-tailed two-sample *t*-test (*t* = 3.942, *df* = 41, *p* = 0.0003). All subjects were male. Basketball players were Chinese national athletes who had experience in competition at national or international tournaments (average training time of 5 days per week with each daily session lasting about 6 hours, for 8.3±2.2 years). The controls were university students without professional training in basketball or any other sports. And in addition to the time of professional basketball training, there is no difference in the time of right-hand use between the athlete group (mean time 30±3.2 hours) and the control group (mean time 32±2.7 hours) in last week (*p* = 0.09). All subjects were right-handed and had no medical history of neurological or psychiatric disease. The protocol was approved by the Research Ethics Committee of Shanghai University of Sport (No.2017106) and all subjects gave written informed consent prior to the experiment.

### Image acquisition

We obtained MRI data using a Siemens Trio Tim 3 T MR scanner. Both DTI and T1-weighted data were acquired using a 12-channel phased-array head coil with the implementation of the parallel imaging scheme of Generalized Auto-calibrating Partially Parallel Acquisitions and with an acceleration factor of 2. DTI data were acquired using a single-shot twice-refocused spin-echo diffusion echo planar imaging (EPI) sequence (repetition time 10000 ms, echo time 92 ms, 64 non-linear diffusion directions with b = 1000 s/mm^2^, an additional volume with b = 0 s/mm^2^, data matrix 128×124, field of view 256 × 248 mm, slice thickness 2 mm, isotropic voxel size 2 mm^3^) and 75 transverse slices without gap covering the whole brain for each. The acquisition time was approximately 12 min for each DTI scan. High resolution 3D isotropic structural images (voxel size 1 mm^3^) were acquired using a T1-weighted magnetization prepared rapid gradient echo sequence (repetition time 1900 ms, echo time 3.44 ms, inversion time 900 ms, flip angle 9°, field of view 256 × 256 mm, slice thickness 1 mm) and 176 sagittal slices covering the whole brain.

### Image preprocessing

We performed data preprocessing in each subject. Both the DTI data and T1-weighted data were visually inspected for apparent artifacts arising from subject motion and instrument malfunction. Distortions in the diffusion tensor images caused by eddy currents and simple head motions were then corrected by applying affine alignment. In particular, EPI distortion induced by the presence of geometrical and intensity distortions along the phase-encode direction, which were caused by field inhomogeneity and concomitant fields, were corrected by registering the first b0 image in each DTI set to its corresponding undistorted T1-weighted image, with a cubic B-spline transformation of knot grid size 10×10×10, partitioning the image space into 2 × 2 × 1.65 cm^3^. After correction, 3D maps of the diffusion tensor and the FA (fractional anisotropy) were calculated. The correction and calculation were performed using FMRIB’s Diffusion Toolbox [21].

### Network construction

We used the automated anatomical labeling template [22], and selected 90 brain regions to construct brain networks for each subject. Each brain region was defined as a node, and each detectable connection between two nodes was defined as an edge. We estimated the topological properties of these brain networks using graph theory [23, 24]. Each subject of 3D structural images was first co-registered to with b = 0 images with linear transformations [25]. The structural images resulted in the diffusion space were mapped to the T1 template of the Montreal Neurological Institute space, using Non-linear Transformations (International Consortium for Brain Mapping 152]. Using the inverse transformation method, the automatic anatomical marker template of the Montreal Neurological Institute was transformed into diffusion space by the nearest neighbor interpolation method. Then we reconstructed the fibers that connect each pair of brain regions in the diffusion space. Based on the Continuous Tracking Algorithm [26], the whole brain fiber was constructed by using the fiber assignment technique. Fiber tracking was stopped at voxels where FA<0.2 or the angle between two eigenvectors of two consecutive voxels connected by the tracking was larger than 35°. The density of the connections between regions (obtained by fiber tractography) is defined as the value of FA. In order to retain more information, and to reflect the heterogeneity in the capacity and intensity of the connections, we used the non-thresholded inter-regional connection density to define the edge. We then obtained a symmetrical connectivity matrix and an anatomical network with weighted edges for each subject. Data preprocessing and network construction were performed using the Pipeline for Analyzing Brain Diffusion Images (PANDA) toolbox [27].

### Network analysis

We analyzed the topological properties of brain networks (global and regional networks) with graph theory [14, 28]. We first analyzed small world network properties proposed originally by Watts and Strogatz (1998) [29] using characteristic path length (L_p_), network clustering coefficient (C_p_), and small-worldness (σ). Additional global network properties including global efficiency (E_glob_) were also examined. (1) Characteristic path length (L_p_) was computed as the average of the shortest path length for all possible edges among nodes in the network. (2) Network clustering coefficient (C_p_) was computed as the average likelihood that the neighbors of a node were interconnected. (3) Small-worldness (σ) was computed as the ratio of C_p_ to the L_p_ with normalization to a null random network. The null networks were generated by randomly connecting to the same number of nodes as in the real network. The mean degree of a null network was set to equal that of the real network. In the present study, the rewiring was repeated 1000 times, and the average value of the null random network was used as the basis to normalize C_p_ and L_p_. Typically, a network is considered to have small-world features if it satisfies the condition of σ >> 1 [29–31].

We then analyzed the regional properties of network measured in the athlete group in comparison to that of the control group using nodal degree, nodal efficiency and betweenness centrality. (1) The nodal degree was computed as the number of the connections linking the node with others. (2) The nodal efficiency was computed as the inverse of the average of the shortest path length in the subgraph defined as the set of nodes that are the neighbors of the node of interest [32]. (3) The betweenness centrality was computed as the fraction of the shortest path between all other pairs of nodes in the network that actually pass through the node [33]. All global and regional parameters were computed using the MATLAB-based Graph Theoretical Network Analysis (GRETNA) toolbox [34].

### Statistical analysis

Between-group (athlete vs. control) differences in the graph-based metrics (global parameters, C_p_, L_p_, E_glob_, σ; regional parameters, degree, efficiency and betweenness) of the anatomical networks were examined by a nonparametric permutation test [16, 35]. Subjects from each group (athlete and novice) were randomly chosen and assigned to two datasets with the same number of subjects in the athlete and control groups. The procedure was repeated for 5000 permutations, resulting in a sampled between-group difference null distribution for each graph-based metric. Finally, we assigned a p-value to the between-group differences by computing the proportion of the differences that exceeded the null distribution values. The threshold for significance was set at p<0.05 with Bonferroni correction for multiple comparisons. In addition, we performed a Pearson’s correlation analysis to test the relationship between the years of training and regional parameters of nodes which show statistically significant differences between groups. The threshold for significance was set at q<0.05 with FDR (False Discovery Rate) correction for multiple comparisons.

### Tract-based spatial statistics (TBSS)

To expand on our examination of network level differences, we lastly investigated group differences in the DTI data at the level of tissue microstructure. All participants’ FA data were aligned into a common space (FMRIB58 FA 1-mm template) using a nonlinear registration tool from FSL, called FNIRT [36], which employs a b-spline representation of the registration warp field [37]. Next, a mean FA image was created and thinned to provide a mean FA skeleton representing the centers of all tracts common to the group. Each participant’s aligned FA data were then projected onto this skeleton and the resulting data were fed into voxel-wise, between-group analysis using FSL Randomise [38]. This method performs a nonparametric two-sample t-test using permutation inference (5,000 permutations) and the threshold-free cluster enhancement (TFCE) test statistic (*p* < 0.01, family-wise error rate corrected). TFCE attempts to locate areas of significant spatial continuity while minimizing problems related to arbitrary cluster threshold and spatial smoothing [39].

## Results

### Global parameters of the brain network

**Fig 1:**
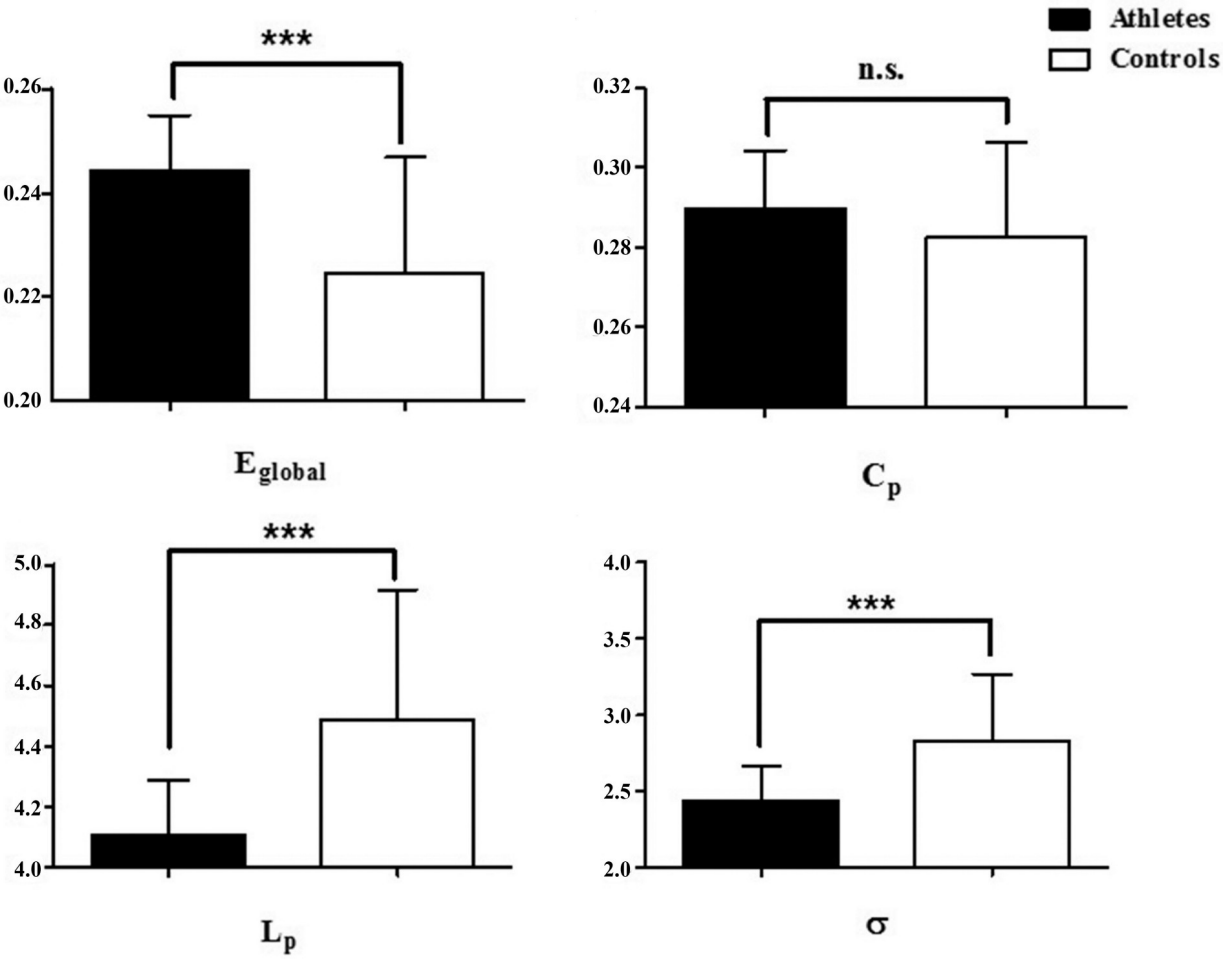
Comparison of global parameters between the athlete group and control group. Black indicates the athlete group while white indicates the control group. The brain network of the athlete group had a higher global efficiency and a lower shortest path coefficient and small-worldness.

Statistical comparisons were performed to detect significant differences in the global parameters and small-world properties of the whole brain anatomical networks between the two groups. We detected significantly higher values of degree and E_glob_ [*t* (41) = 3.735, *p* = 0.0003] and but significantly lower values of L_p_ [*t* (41) = 3.933, *p* = 0.0002] and σ [*t* (41) = 3.662, *p* = 0.0004] and σ [*t* (41) = 3.662, *p* = 0.0004]in elite athlete group. However, we found no statistically significant differences in the value of C_p_ (*t* (41) = 1.235, *p* = 0.009) (Fig 1). The results showed that compared to the control group, the brain networks were denser and more random in the athlete group. These findings are consistent with previous studies [16]. We found that the measured parameters for control subjects varied from those reported in research published by Wang and his colleagues in 2013 [16]. The measured parameters also seemed to differ from those in those reported in research published by Iturria-Medina and his colleagues in 2008 [40]. We think the reason for this discrepancy lied in the fact that in Wang and Iturria-Medina’s studies, they constructed a binary network without weighted edges. Nevertheless, we constructed a weighted network, which the FA value between a pair of nodes was the weight value of edges. It would cause the discrepancy of the measured parameters that whether contructing the binary or weighted network, and choosing a different indices for the weight [30, 41–42].

### Regional parameters of the brain network

**Fig 2:**
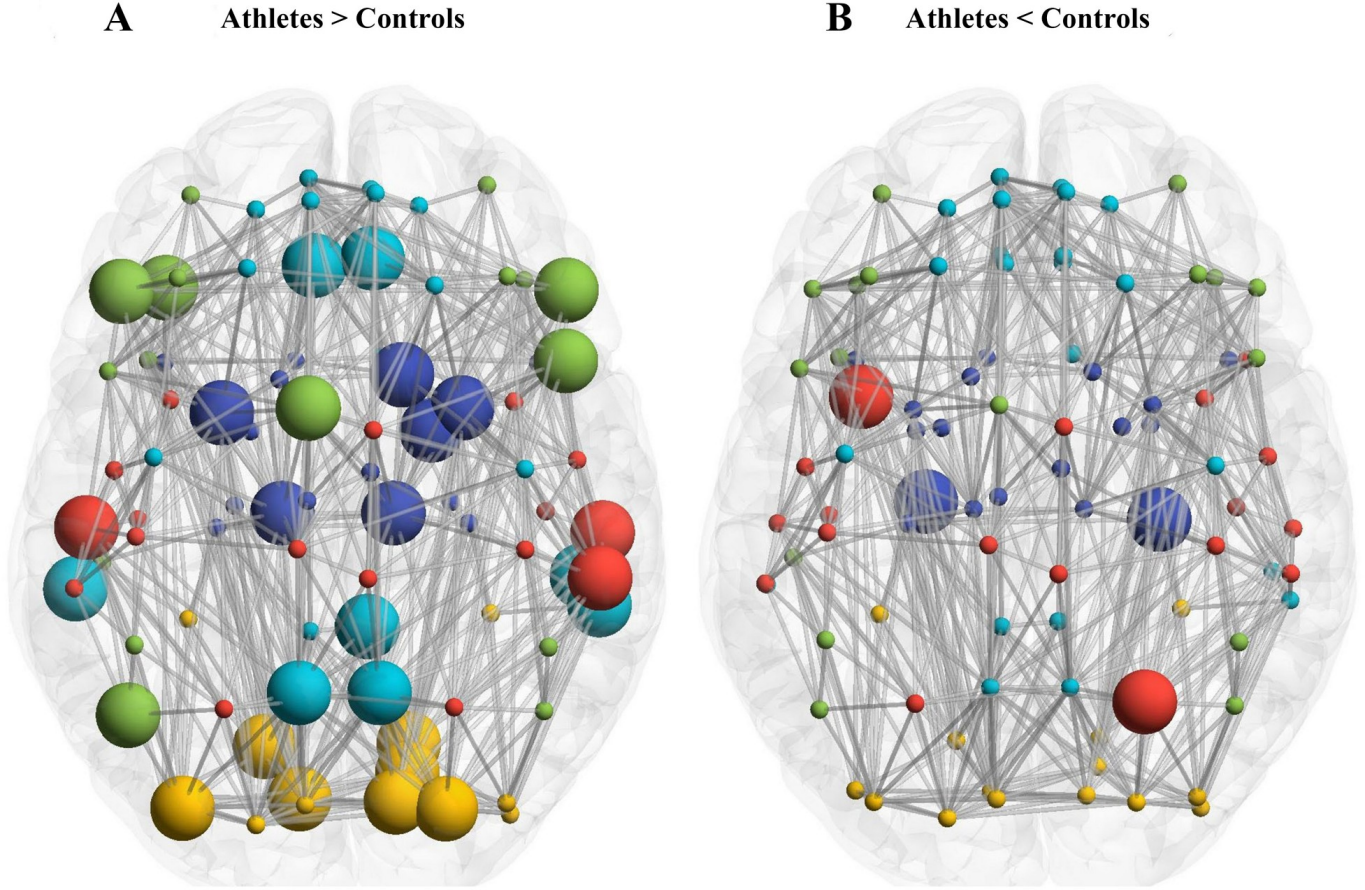
Comparison of regional parameters between athlete and control groups. A shows the bigger node size of brain networks in athletes group with higher regional parameters compared to the control group. B shows the bigger node size of brain networks in the control group with higher regional parameters relative to the athlete group. Red nodes indicate the sensorimotor network while yellow nodes indicate the visual network, green nodes the attention network, light blue nodes the default-mode network, and the dark blue nodes the limbic/subcortex network.

Table 1 lists the results of statistical comparisons of the regional parameters of the anatomical networks between the two groups (p<0.01). We found 14 brain regions with significantly higher degrees, 29 brain regions with significantly higher regional efficiency, three brain regions with significantly higher between-ness centrality, and four brain regions with significantly lower between-ness centrality in the anatomical networks of the athlete group compared to the controls. There were 34 brain regions with significant differences in regional parameters between the two groups. Among them, the regions with higher regional parameters in the athlete group were located in the visual, default-mode, and attention networks; while the rest of the regions were located in the sensorimotor and limbic/subcortical network (Fig 2).

**Table 1:** Changes in regional parameters in basketball players.

Subnetwork	Brain areas	Degree	Efficiency	Betweenness
Attention	IFGoperc.R	↑	↑	↑
	IFGtriang.L	−	↑	−
	IFGtriang.R	↑	↑	−
	ORBinf.L	↑	↑	−
	SMA.L	−	↑	−
	ANG.L	−	↑	−
Sensorimotor	INS.L	−	−	↓
	SPG.R	−	−	↓
	SMG.R	−	↑	−
	STG.L	−	↑	−
	STG.R	↑	↑	−
Default-mode	ACG.L	−	↑	−
	ACG.R	−	↑	−
	PCG.R	↑	↑	−
	PCUN.L	−	↑	−
	PCUN.R	↑	↑	−
	MTG.L	−	↑	−
	MTG.R	−	↑	−
	ITG.R	−	−	↑
Visual	CAL.L	↑	↑	−
	CAL.R	↑	↑	−
	CUN.R	↑	↑	−
	LING.L	↑	↑	−
	LING.R	−	↑	−
	SOG.R	↑	↑	−
	MOG.L	−	↑	−
Limbic/Subcortical	HIP.R	−	−	↓
	PHG.L	−	−	↓
	CAU.R	−	↑	−
	PUT.L	↑	↑	−
	PUT.R	−	↑	−
	PAL.R	↑	↑	↑
	THA.L	↑	↑	−
	THA.R	↑	↑	−

↑ showed that the value of regional parameters of nodes in athletes brain network were higher than those nodes in controls brain network while ↓ showed that the value of regional parameters of nodes in athletes brain network were lower than those nodes in controls brain network. IFGoperc, inferior frontal gyrus, opercular part; IFGtriang, inferior frontal gyrus, triangular part; ORBinf, inferior frontal gyrus, orbital part; SMA, supplementary motor area; ANG, angular gyrus; INS, insula; SPG, superior parietal gyurs; SMG, supramarginal gyrus; STG, superior temporal gyurs; ACG, anterior cingulated and paracingulated gyrus; PCG, posterior cingulate gyrus; PCUN, precuneus; MTG, middle temporal gyrus; ITG, inferior temporal gyrus; CAL, calcarine; CUN, cuneus; LING, lingual gyrus; SOG, superior occipital gyrus; MOG middle occipital gyrus; HIP, hippocampus; PHG, parahippocampal gyrus; CAU, caudate; PUT, putamen; PAL, pallidum; THA, thalamus.

### Correlation between years of training and regional parameters

**Fig 3:**
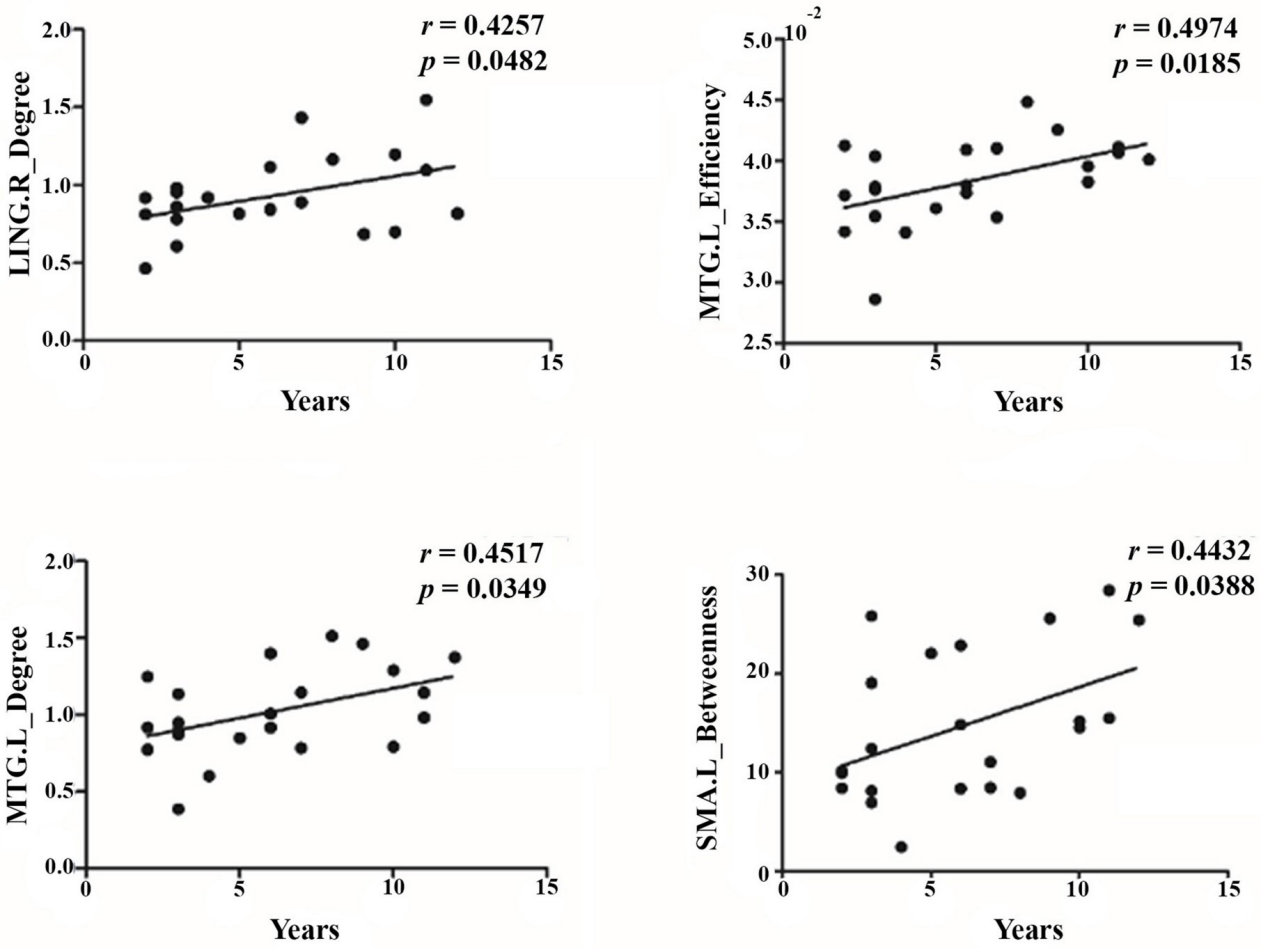
The correlation between regional parameters and years of training. SMA: supplementary motor area; MTG: middle temporal gyrus; LING: lingual gyrus. The results show that the regional parameters of right SMA, left MTG and right LING are positively correlated to the number of years of training.

We analyzed the correlation between the years of training and the three regional parameters (Degree, Efficiency and Betweenness) of all 90 nodes in the brain network of athlete group and performed the FDR correction for multiple comparisons. Fig 3 reveals a tendency for the regional parameters to change with years of training, of which the regional parameters of the left l middle temporal gyrus (Efficiency, *q* = 0.0432; Degree, *q* = 0.0489), right lingual gyrus (*q* = 0.0482), and left supplementary motor (*q* = 0.0453)area have positive correlations with years of training (Fig 3).

### Tract-based spatial statistics (TBSS)

**Fig 4:**
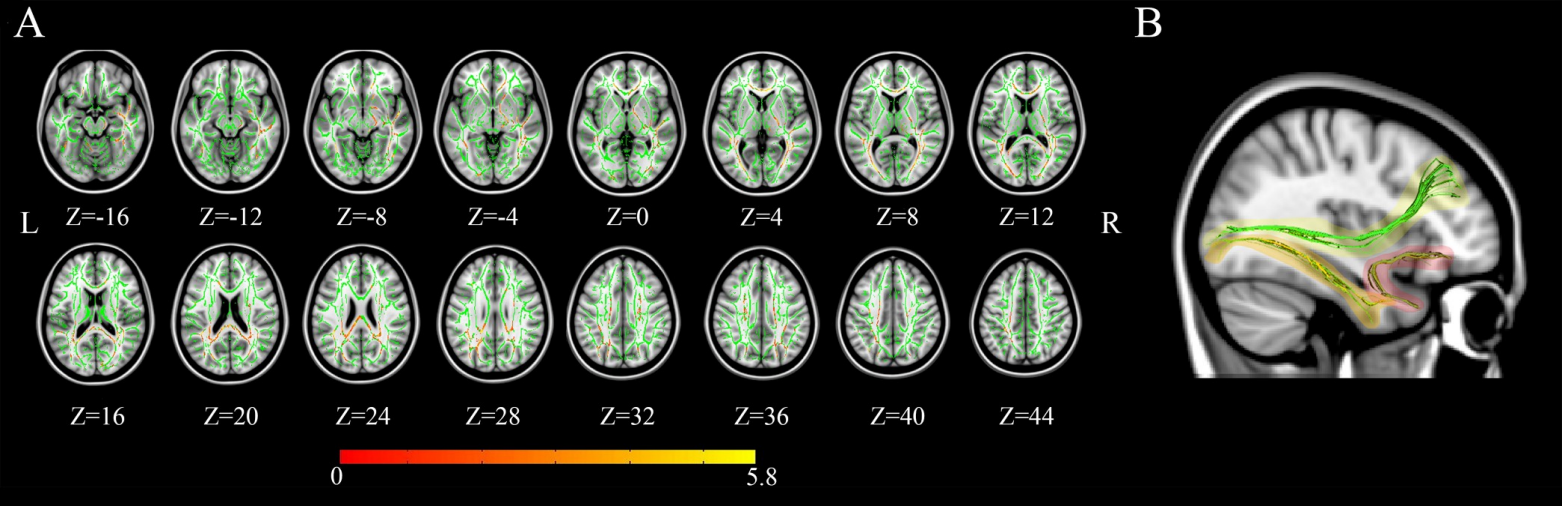
Tissue microstructure result of white matter. (A) Green denotes the white matter skeleton and red denoted the areas with significant bigger fractional anisotropy. (B) The reconstructed streamlines are shown for the major three nerve fiber bundles in the brain. Yellow denoted the inferior fronto-occipital fasciculus connecting occipital and limbic system. Orange denoted the inferior longitudinal fasciculus connecting occipital and temporal. Red denoted the uncinated fasciculus connecting orbitofrontal and basal ganglia.

The TBSS was conducted to investigate tissue microstructural features that may provide anatomical explanations for the distribution of network connectivity differences. The elite athlete group demonstrated significantly higher FA (*p* < 0.01, corrected) compared with controls in multiple regions throughout the brain (Table 2 and Fig 4A). Nerve fiber bundles showing FA differences were mainly located on the inferior longitudinal fasciculus, uncinate fasciculus, and inferior fronto-occipital fasciculus, which are among the major long range fiber tracts connecting distant cortical areas [43] (Fig 4B). No significantly higher FA (p <0.01) in controls were found compared with elite athlete group.

**Table 2:** Fractional anisotropy data for peak voxels of athletes > novices.

N of voxel	*t*-value	x	y	z	Label
18063	5.78	50	-6	-8	Right inferior longitudinal fasciculus
1523	5.2	13	33	-8	Forceps minor
					Right uncinate fasciculus
134	5.78	37	-81	-3	Right inferior longitudinal fasciculus
125	3.43	-18	-91	4	Forceps minor
					Left inferior fronto-occipital fasciculus
					Left inferior longitudinal fasciculus
50	2.07	26	-23	-3	Right optic radiation
40	4.19	-32	28	25	Left anterior thalamic radiation
37	3.5	-32	-14	41	Left superior longitudinal fasciculus
32	2.92	10	-85	15	Forceps major
21	1.76	17	-53	27	Callosal body
20	2.37	35	-62	-2	Right inferior longitudinal fasciculus
					Right inferior fronto-occipital fasciculus
11	2.97	-47	-1	21	Left superior longitudinal fasciculus

Peak voxel locations included bilateral inferior longitudinal fasciculus, bilateral inferior fronto-occipital fasciculus, left superior longitudinal fasciculus, left anterior thalamic radiation, forceps minor and major, right uncinate fasciculus and right optic radiation. Co-ordinates are shown in MNI (Montreal Neurological Institute) space.

## Discussion

In this study, we investigated the changes in structural networks of brain white matter between people with professional skills and controls. The results show that global network parameters of the two groups are different, and the changes in network properties are predominantly in the visual, default-mode, and attention networks. Furthermore, the main changes in visual regions were related to the years of training, and showed that motor skill learning can improve the ability of our brains to control behavior, make decisions more effectively, and switch the focus of attention more accurately.

### Open skill and brain subnetworks

The results of the network analysis at both global and regional levels are consistent with the results by Wang et al (2013). First, at the whole brain level, we found significantly greater values of E_glob_, but significantly lower values of L_P_ and σ in the anatomical networks of the athlete group compared to the controls. The anatomical networks of both groups exhibited small-world properties, a finding which is consistent with previous studies [16]. Small-worldness supports both integrated and distributed information processing and maximizes the efficiency of propagating information at a relatively low cost [44]. However, the anatomical networks of the athlete group were more random, with a lower value of L_P_, indicating that the connections among distant brain regions may be tighter after prolonged training in professional skills. The obvious changes may point to the involvement of distributed cortex-subcortex connections when people learn professional skills. The lower value of Lambda may be associated with higher efficiency among nearby brain regions [45, 46]. Repetitive skill learning renders motor processes more automatic and increases the accuracy of motor performance. Based on our results, it is reasonable to assume that after long-term professional skills training, the athlete group could transfer and integrate the local information automatically. In the athlete group, the random network properties ensured that the connections among distant brain areas were faster and more direct, thereby increasing the effectiveness of switching between functional areas.

At the regional level, we found that the attentional and default-mode networks were still the major subnetworks that were changed, which is similar to the results reported by Wang et al (2013). We believe that these results may partly be caused by the brain mechanisms underlying the acquisition of open and closed skills. The regions of the attentional network are mainly located in the dorsal attention system, which comprises the parietal cortex and superior frontal gyrus. These regions relate to a directional stimulus, target selection, and preparation reaction. The dorsal attention network is responsible for the coordination of complex motor functions and control of motor plans [47]. It is considered a bridge between the central executive network and default-mode network [48]. A clinical study showed that the frontal-parietal network may contribute to effectively coordinate complex visual movement [49]. In addition, the default-mode network is related to episodic memory retrieval [50–53] and self-reflection [54]. Enhancement of default-mode network activity may help to extract episodic memories [55]. In addition, another important function of the default-mode network is to monitor the external environment [56–58]. The changed default-mode regions of our study were in the so-called “dorsal medial prefrontal cortex subsystem.” The function of this subsystem is to guide and drive behavior when information about external stimuli is processed. This may be generated by the interaction of the default-mode network and subcortical regions or the internal mental processing functions of two different subsystems of the default-mode network. The dorsal medial prefrontal system may reflect the state of an individual’s mind induced by external stimulation; for example, when individuals need to identify a particular social context (such as a basketball scene). In contrast, the medial temporal lobe system may integrate the existing experience of the past and thus contribute to goal-directed behavior. Based on the consistency of our results with those of Wang et al. (2013), we propose that the two networks are the basis of the acquisition of different types of skills.

Consistent with our hypothesis, the visual network plays an important role in the acquisition of open skills. It is believed that there are two visual pathways in the brain. The first pathway is the ventral pathway, which transmits information to the temporal cortex with V4 as the center. The second pathway is the dorsal pathway, which transmits information to the parietal cortex with MT (middle temporal / V5) as the center [59]. The ventral pathway is responsible for identifying and recognizing objects, whereas the dorsal pathway is responsible for helping the motion system detect and use objects, and is also involved in behavioral control [60]. The result of the group comparison revealed that the regional parameters of some brain areas showed significant differences. These areas included the calcarine area, cuneus, lingual gyrus, and middle occipital gyrus. Collectively, these areas are part of the peripheral striatum. Research on monkeys has revealed that the peripheral striatum is a collection of different areas involved in processing visual information and multiple representations of the visual scene [61]. Therefore, it appears to be the differentiation point of the two visual pathways. The increase in regional parameters of brain areas in the peripheral striatum suggests that after long-term motor skill learning, the brain areas which used to distinguish different visual information are more important. They can therefore characterize visual input more quickly and transmit the information to the relevant visual pathways for visual processing.

### The relationship between regional parameters and years of training

The correlation analysis of regional parameters and years of training showed that the between-ness of the left supplementary motor area as well as the degree of right lingual gyrus and left middle temporal gyrus, increased with years of training. Notably, these changes were specific to years of training. The supplementary motor area is important for motor planning and organizing a rapid motor sequence based on a specific order [62–64] also reported that the supplementary motor area can suppress habitual behavior to engage in other activities. As part of the ventral visual pathway, the lingual gyrus is related to complex image coding. Machielsen et al. (2000) [65] considered the lingual gyrus to be responsible for memory and recognition of neutral pictures. Furthermore, the lingual gyrus is involved in selective visual attention. Mangun et al. (1998) [66] found that the lingual gyrus is highly activated when remembering the characteristics of objects in a particular visual field and ignoring objects in other visual fields. Based on the results of our correlation analysis, we propose that in the athlete group, the years of training of all subjects are decided by acquired factors. As such, the changes in regional parameters of brain areas related to years of training may be induced by the learning interventions. We speculate that with the increase of the years of training, the importance of supplementary motor area which was responsible for perceiving others’ and own actions and position automatically and make a decision as well as lingual gyrus, which was responsible for selectively processing visual information as well as controlling habitual behavior and fast motor sequences, are improved.

It is worth noting that the regional parameters of the left middle temporal gyrus showed a significant positive correlation with the years of training. It seems that the left middle temporal gyrus plays a critical role in motor skill learning. The middle temporal gyrus is important for semantic information processing and lexical representation and storage [67]. Together with the rostral supplementary motor area, the middle temporal gyrus is responsible for monitoring internal speech [68]. As the hub of the dorsal temporal lobe system of the default-mode network, the middle temporal gyrus is related to the complex characteristics of objects and high-level cognitive functions such as semantic memory and visual perception of information [69, 70]. Kim et al. (2008) [71] found that when world-class archers focused on targets, their middle temporal gyri were significantly activated. Draganski et al. (2004) [3] also found that the volume of grey matter of bilateral temporal gyri of acrobats increased significantly. These studies show that the middle temporal gyrus may be able to promote the transmission between visual information and motion perception, which helps to coordinate visual motor skill learning and performance. Research using PET (Position Emission Tomography) on semantic paralysis and normal subjects found that the anterior middle temporal gyrus is not only a semantic processing center, but is also responsible for integrating visual, auditory, motor, and functional information, as well as vocabulary and concepts, to form high-level amodal conceptual representations [72–74]. With the increase of the years of training, the importance of middle temporal gyrus, which is responsible for interpreting and abstracting the science scene, extracting key information, and making decisions accurately, has been improved. The enhancement of these functions promotes superiority when subjects in the athlete group perform professional skills.

## Limitation

Although graph theory are applied increasingly more to detect brain plasticity, few studies have focused on the influence of motor skills learning on brain networks. Wang and his colleagues’ exploration of the structural and functional network characteristics of the brain in World Gymnastics Champion is an excellent achievement in this field. Our research draws on the methods of Wang and his colleagues and attempts to explore the impact of open skill learning on brain networks. We believe that our research complements the field of motor skills learning that affects the brain network.

In this study, we regarded basketball as a representative of the open skills. However, the results did not fully represent the characteristics of the open skills. Closed skills (Gentile, 1972) are those without environmental uncertainty during planning (e.g. repeating the same motion without variation in external conditions). Open skills are skills with environmental uncertainty (e.g. those that require ongoing sensory feedback). Schmidt [75] suggested that this difference is not useful, because, for example, once a decision is made to produce a component movement, the response cannot be changed for some small interval around 200ms. Also, if warm up and fatigue in muscles are considered as components of environmental uncertainty, there is also (at most) a limited time period over which even highly repetitive skills may be considered closed. Moreover, our results are consistent with the results of Wang, et al. (2013) support the viewpoint that closed versus open skills produce similar changes in brain connectivity as measured by DTI. In the future, it is valuable to test the differences between open and closed skill group.

Although the study found that the node parameters of the visual, attention and sensorimotor areas of brain network in athletes group were improved, and these improvements were related to the duration of the basketball training. But this did not necessarily mean that basketball players had the better ability of visual, attention and movement related ability. We need to do further behavioral experiments related to these functions and correlate the data of behavioral experiments with the brain network data in order to finally get a conclusion.

## Conclusions

In summary, we constructed brain anatomical networks for the athlete group with professional skills and controls using DTI and deterministic tractography. Using a graphical analysis approach, we found that the ability of long-distance information transmission in the brain was stronger, and the network was more random in the athlete group. Moreover, the importance of attention, sensorimotor, default-mode and visual function regions in the network was improved in the athlete group. More importantly, the main factor leading to enhancement of these functions is induced by acquired motor learning. We believe that motor skill learning can improve the ability of our brains to control behavior, make decisions more effectively, and switch the focus of attention more accurately. Our results provide insight into the mechanisms underlying brain plasticity and may help to develop the brain’s potential for change as well as to treat brain injury.

## Supporting information

S1 Compressed Raw Data Rawdata.rar. (RAR)
